# Physical Activity and Sedentary Behavior in Relation to Esophageal and Gastric Cancers in the NIH-AARP Cohort

**DOI:** 10.1371/journal.pone.0084805

**Published:** 2013-12-19

**Authors:** Michael B. Cook, Charles E. Matthews, Munira Z. Gunja, Zaynah Abid, Neal D. Freedman, Christian C. Abnet

**Affiliations:** Division of Cancer Epidemiology and Genetics, National Cancer Institute, Bethesda, Maryland, United States of America; Kagoshima University Graduate School of Medical and Dental Sciences, Japan

## Abstract

**Introduction:**

Body mass index is known to be positively associated with an increased risk of adenocarcinomas of the esophagus, yet there is there limited evidence on whether physical activity or sedentary behavior affects risk of histology- and site-specific upper gastrointestinal cancers. We used the NIH-AARP Diet and Health Study to assess these exposures in relation to esophageal adenocarcinoma (EA), esophageal squamous cell carcinoma (ESCC), gastric cardia adenocarcinoma (GCA), and gastric non-cardia adenocarcinoma (GNCA).

**Methods:**

Self-administered questionnaires were used to elicit physical activity and sedentary behavior exposures at various age periods. Cohort members were followed via linkage to the US Postal Service National Change of Address database, the Social Security Administration Death Master File, and the National Death Index. Cox proportional hazards regression models were used to estimate hazard ratios (HR) and 95 percent confidence intervals (95%CI)

**Results:**

During 4.8 million person years, there were a total of 215 incident ESCCs, 631 EAs, 453 GCAs, and 501 GNCAs for analysis. Strenuous physical activity in the last 12 months (HR_*>5 times/week vs. never*_=0.58, 95%CI: 0.39, 0.88) and typical physical activity and sports during ages 15–18 years (p for trend=0.01) were each inversely associated with GNCA risk. Increased sedentary behavior was inversely associated with EA (HR_*5–6 hrs/day vs. <1 hr*_=0.57, 95%CI: 0.36, 0.92). There was no evidence that BMI was a confounder or effect modifier of any relationship. After adjustment for multiple testing, none of these results were deemed to be statistically significant at p<0.05.

**Conclusions:**

We find evidence for an inverse association between physical activity and GNCA risk. Associations between body mass index and adenocarcinomas of the esophagus do not appear to be related to physical activity and sedentary behavior.

## Introduction

Approximately 25% of all cancers worldwide can be attributed to excess weight and a sedentary lifestyle [1]. Over the last few decades in the United States, levels of excess weight have been increasing [2] and occupational physical activity decreasing [3]. A concurrent observation has been a steep increase in the incidence rate of esophageal adenocarcinoma of over 650% [4]. Some of this increase in incidence may be attributable to obesity and sedentary lifestyle; indeed, a previous publication by our group, using NIH-AARP data from the baseline questionnaire, indicated that increased physical activity may be protective against adenocarcinomas of the esophagus and stomach [5]. The NIH-AARP prospective cohort study now has more comprehensive exposure assessment of both physical activity and sedentary behavior. Coupled with the updated follow-up for cancer outcomes through calendar year 2006, we assessed these exposures in relation to the risk of esophageal and gastric malignancies.

## Methods

For this analysis of physical activity and sedentary behavior in the NIH-AARP Diet and Health Study, we prepared two overlapping analytic cohorts in order to assess exposures included in the baseline questionnaire and exposures included in the subsequent risk factor questionnaire in relation to the risk of esophageal and gastric malignancies.

### Baseline questionnaire cohort

The NIH-AARP Diet and Health study cohort consists of men and women aged 50–71 years, at the time of recruitment for the study. Participants resided in one of six states (California, Florida, Louisiana, New Jersey, North Carolina, or Pennsylvania) or one of two metropolitan areas (Atlanta, Georgia or Detroit, Michigan). In 1995–1996, a self-administered questionnaire, assessing medical history, diet, and demographic characteristics, was completed by 566,398 AARP members. 

For analyses of baseline questionnaire exposures, we excluded individuals who: had questionnaire responses completed by a proxy (n=15,760); self-reported a prior cancer diagnosis (n=49,318) or end-stage renal disease (n=997); had a registry confirmed cancer diagnosis prior to study entry (n=1,903); and who had only a death report of an upper gastrointestinal tract cancer (n=202). We also excluded those with extreme box-cox-transformed energy intake values (n=4,397) and those with zero follow-up time (n=19). Our final analytic cohort, for baseline questionnaire exposures, consisted of 493,802 participants. 

Herein, we analyze the baseline questionnaire questions that ascertained: the level of current routine daily activities at baseline; the frequency of strenuous physical activity in the last 12 months; and the frequency of physical activity and sports during ages 15 to 18 years. The questions and potential responses used to elicit these exposures are shown in Table 1.

**Table 1:** Sedentary activity and physical activity exposures ascertained on the baseline and risk factor questionnaires.

**Questionnaire **		**Question**		**Responses**	
Baseline		Currently, which of the following best describes your daily routine at work? If you do not work at a job, select the response that best describes your routine throughout the day. Do not include the time you spend exercising or playing sports.		You sit during the day and do not walk around very much / You sit much of the day, but also walk around afair amount / You stand or walk around a lot during the day, but do not have to carry or lift things very often / You lift or carry light loads, or have to climb stairs or hills often / You do heavy work or carry heavy loads	
		During a typical month in the past 12 months, how often did you participate in physical activities at work or home, including exercise, sports, and activities such as carrying heavy loads? Only include periods of physical activities that lasted at least 20 minutes and caused increases in breathing or heart rate, or caused you to work up a sweat.		Never / Rarely / 1–3 times per month / 1–2 times per week / 3–4 times per week / 5 or more times per week	
		Think back in time to when you were around the ages of 15 to 18 years old. Back then, about how often did you participate in physical activities or sports during a typical month?		Never / Rarely / 1–3 times per month / 1–2 times per week / 3–4 times per week / 5 or more times per week	
Risk Factor		Read the list of examples of moderate and vigorous activities in the box below. Think back to the ages and time listed in the table below. Mark the circles that best describe how often you participated in moderate and vigorous activities at the ages and time listed. Do not include activities that you reported in questions 48–51 on page 13 [job-related MVPA]. How often did you participate in moderate and vigorous activities at the following ages?		Ages 15–18, 19–29, 35–39, and in the past ten years. Never / Rarely / Weekly but less than 1 hour per week / 1–3 hours per week / 4–7 hours per week / more than 7 hours per week	
		During a typical 24-hour period over the past 12 months, how much time did you spend watching television or videos?		None / Less than 1 hour / 1–2 hours / 3–4 hours / 5–6 hours / 7–8 hours / 9 or more hours	
		During a typical 24-hour period over the past 12 months, how many hours did you spend sitting:		Less than 3 hours / 3–4 hours / 5–6 hours / 7–8 hours / 9 or more hours	

### Risk factor questionnaire

Six months after completion of the baseline questionnaire, a risk factor questionnaire, which asked about additional exposures including strenuous physical activity and sedentary behavior, was mailed to the 542,095 who had completed the baseline questionnaire and had not self-reported colon, breast, or prostate cancer. The risk factor questionnaire was satisfactorily completed by 337,076 of these individuals. From this there were 1,619 who died before entry, 547 who moved before entry, and 3 individuals who withdrawn, all of who were deemed ineligible. There remained 334,905 eligible participants for the risk factor questionnaire cohort.

From this eligible cohort, we excluded individuals who: had baseline or risk factor questionnaire completed by a proxy (n=10,383); self-reported a prior diagnosis of cancer (n=16,082); had a registry confirmed cancer diagnosis prior to study entry (n=2,775); had only a death report of an upper gastrointestinal tract cancer (n=118); had extreme box-cox-transformed energy intake values (n=2,503); and had zero follow-up time (n=11). Our final analytic cohort for risk factor questionnaire exposures consisted of 303,033 participants. 

Herein, we analyze the following exposures ascertained through the risk factor questionnaire: level of recreational moderate-vigorous physical activity in the past ten years and in four earlier life-periods (15–18, 19–29, 35–39 yrs); the amount of time spent watching television or video in a typical 24 hour period during the last 12 months; and amount of time sitting in a typical 24 hour period during the last 12 months. The specific questions and potential responses from the risk factor questionnaire are shown in Table 1.

The study was approved by the Special Studies Institutional Review Board of the U.S. National Cancer Institute, NIH. All participants gave informed consent by completing and returning the questionnaires. 

### Cohort Follow-up and ascertainment of cancer diagnoses

Cohort members were followed by linkage to the US Postal Service National Change of Address database, through processing of undeliverable mail, address change services, and direct contact with participants. Vital status was determined through linkage with the Social Security Administration Death Master File [6,7], and determinations of vital status and causes of death were made by using the National Death Index [8]. The primary endpoints for our analysis were esophageal squamous cell carcinoma (ESCC), esophageal adenocarcinoma (EA), gastric cardia adenocarcinoma (GCA), and gastric non-cardia adenocarcinoma (GNCA). 

### Statistical Analysis

We used multivariable Cox proportional hazards regression models to assess physical activity and sedentary behavior exposures in relation to the risk of ESCC, EA, GCA, and GNCA. Follow-up time started from the date the relevant questionnaire was scanned. Individuals were right-censored at date of death, date of a none-outcome cancer diagnosis, date of last follow-up prior to loss of follow-up, or the end of follow-up (31 December 2006), whichever occurred first. Adjusted models included the baseline covariates age (continuous; baseline/risk factor questionnaires), sex, body mass index (BMI; categorical: <18.5, 18.5–<25, 25–<30, 30–<35, ≥35, missing), education (categorical: <11 years, high school graduate, vocational/some college, college graduate, post graduate, missing), ethnicity (categorical: non-Hispanic white, non-Hispanic black, Hispanic, Asian/Pacific Islander, American Indian/Alaskan Native, Missing), perceived health status (categorical: good, poor, missing), alcohol consumption (categorical: none, 0 drink/day, 1-3 drinks/day, >3 drinks/day, missing), cigarette smoking (categorical: never, quit/≤20 cigs/day, quit/20 cigs/day, current/≤20 cigs/day, current/20 cigs/day, missing), fruit consumption (continuous) and vegetable consumption (continuous). Exposures were ascertained from the baseline questionnaire and were current measures at that time, unless other specified. Exposures ascertained from the risk factor questionnaire that were included in risk factor questionnaire exposure models included aspirin (dichotomous: no, yes, missing), ibuprofen (dichotomous: no, yes, missing) and antacids (dichotomous: no, yes, missing). All dietary measures were ascertained through use of a 124 food items on the baseline questionnaire. Given the possibility that BMI could be on a causal pathway of these exposures in relation to disease, we also conducted models without adjustment for BMI. In addition, to investigate potential effect-modification of physical activity and sedentary behavior exposures by BMI, we also stratified all analyses by BMI using the categories <25, 25 to <=30, and >30. A false-discovery rate method was used to control the type I error [9]. We also conducted a lag analysis whereby upper gastrointestinal cancer cases were split into three equal groups based on follow-up time, with the first two groups being sequentially omitted from the analysis. We also conducted mutually adjusted models for typical recreational moderate-vigorous physical activity at multiple age periods (ages 15–18, 19–29, 35–39, and last ten years), as well as for sedentary behavior and physical activity (i.e., typical recreational moderate-vigorous physical activity with typical number of hours per day watching TV in the last 12 months, and separately with typical number of hours per day sitting during the last 12 months; current daily routine at work with strenuous physical activity during last 12 months). The proportional hazards assumption was assessed using a global test comparing the main models to a model with inclusion of exposure-survival time interaction parameters, and was satisfied for all models. All analyses were conducted using SAS 9.1 (SAS Institute Inc) and all statistical tests were 2-sided with p<0.05 deemed as statistically significant. 

## Results

The demographic characteristics of each of the analytic cohorts are shown in Table 2. The cohorts were very similar, which is not surprising given that the Risk Factor cohort is nested within the Baseline cohort. As can be seen for the baseline questionnaire analytic cohort, the average age at questionnaire completion was 62 years, of which 60% were men and 91% were non-Hispanic white. Forty-two percent of responders were overweight and 21% were obese. Fourteen percent self-reported as current cigarette smokers and 8% reported consuming more than three alcoholic drinks per day. The perceived health status was good for a vast majority with only 12% reporting poor health, and 39% of individuals were college graduates. 

**Table 2:** Distribution of covariates among each analytic cohort from the NIH-AARP Diet and Health Study.

**Variable**		**Baseline Questionnaire**											**Risk Factor Questionnaire**						
		**Analytic Cohort**			**Incident Cases**								**Analytic Cohort**			**Incident Cases**			
					**ESCC**	**EA**		**GCA**	**GNCA**							**ESCC**	**EA**	**GCA**	**GNCA**
		**(n=493,802)**			**(n=215)**	**(n=631)**		**(n=453)**	**(n=501)**				**(n=303,033)**			**(n=128)**	**(n=377)**	**(n=255)**	**n=(277)**
Total follow-up time in years / Time to event in years, median (25th, 75th percentiles)		4,795,319			4.87(2.60, 7.15)	6.24(3.70, 8.78)		5.34(2.80, 7.89)	5.74(2.90, 8.59)				2,797,622			4.79(2.80, 6.52)	5.90(2.90, 7.94)	5.02(2.72, 7.61)	5.65(2.38, 7.77)
Age in years, mean (s.d)		62.0 (5.4)			63.49 (4.98)	63.59 (4.99)		63.57 (5.00)	64.57 (4.65)				62.85 (5.31)			64.81 (4.66)	64.43 (4.90)	64.53 (4.78)	65.32 (4.71)
Fruit intake, mean (s.d) (servings/1000 kcal/day)		1.73 (1.17)			1.33 (1.01)	1.5 (1.09)		1.57 (1.21)	1.76 (1.28)				1.74 (1.16)			1.40 (0.99)	1.56 (1.17)	1.59 (1.23)	1.71 (1.24)
Vegetables, mean (s.d) (servings/1000 kcal/day)		2.26 (1.16)			2.26 (1.23)	2.14 (1.21)		2.11 (0.97)	2.15 (1.11)				2.28 (1.16)			2.37 (1.34)	2.17 (1.12)	2.15 (1.05)	2.03 (0.91)
Sex, %																			
	Male	59.65			66.51	92.71		87.86	71.06				58.33			64.84	92.57	87.06	73.65
	Female	40.35			33.49	7.29		12.14	28.94				41.67			35.16	7.43	12.94	26.35
Ethnicity, %																			
	Non-Hispanic White	91.24			88.37	96.67		94.92	79.24				92.57			89.84	97.88	94.12	82.67
	Non-Hispanic Black	3.87			6.51	0.79		1.99	8.98				3.28			7.03	0.8	2.35	7.22
	Hispanic	1.91			1.4	1.11		1.1	5.79				1.62			0.78	0.53	1.57	4.69
	Asian/Pacific Islander or American Indian/Alaskan Native	1.65			1.86	0.79		1.55	3.99				1.42			0	0.53	1.57	2.53
Body mass index, %																			
	<18.5	0.93			5.58	0.48		0.44	1.2				0.95			5.47	0.53	0.78	1.08
	18.5-<25	33.53			46.98	19.97		22.52	30.34				35.22			48.44	20.42	23.14	30.69
	25-<30	41.56			29.77	48.02		42.6	41.12				41.18			29.69	45.09	41.18	41.88
	30-<35	15.36			11.63	22.19		21.85	18.36				14.75			11.72	26.26	22.75	18.05
	>=35	6.09			1.86	7.29		9.27	5.39				5.76			1.56	6.37	9.02	5.05
Cigarette smoking, %																			
	Never	35.11			13.02	16.16		15.67	27.94				35.9			14.84	14.06	16.08	27.8
	Quit, <=20 cigs/day	26.61			14.42	25.2		24.72	24.35				27.06			16.41	26.79	25.1	22.74
	Quit, >20 cigs/day	20.77			30.7	35.97		36.42	27.35				21.11			34.38	39.52	38.43	29.6
	Current, <=20 cigs/day	8.87			21.86	8.72		9.05	9.98				8.17			19.53	8.22	9.02	7.94
	Current, >20 cigs/day	4.85			13.95	9.35		7.51	5.19				4.44			11.72	6.9	5.88	5.78
Alcohol consumption, %																			
	None	24.02			20	19.18		24.28	25.95				23.08			17.97	17.24	25.1	28.16
	0-1 drinks/day	52.92			29.3	50.71		47.24	48.3				53.41			32.03	52.52	43.92	46.93
	1-3 drinks/day	15.13			20.47	17.75		15.89	17.96				15.69			20.31	18.3	17.25	16.97
	>3 drinks/day	7.52			30.23	12.04		12.36	6.99				7.51			29.69	11.67	13.73	6.5
Perceived health status, %																			
	Good	86.49			75.35	83.84		80.35	86.03				87.67			73.44	85.68	80.78	86.64
	Poor	12.02			21.86	14.9		17.88	11.58				11.04			24.22	13	17.25	10.47
Education, %																			
	<12 years	5.96			10.23	7.92		9.93	10.38				4.9			7.81	6.37	7.06	7.94
	High School graduate	19.54			15.81	19.02		19.21	21.96				18.5			15.63	15.92	20.39	19.13
	Vocational, Some college	33.03			34.88	35.66		32.23	31.14				33.12			32.81	37.4	27.84	32.85
	College graduate	18.82			20.93	17.43		17.66	16.57				19.7			23.44	21.49	21.57	19.49
	Post graduate	19.74			14.42	16.01		16.78	15.17				21.32			18.75	15.92	19.61	16.25
Aspirin, %																			
	No	-			-	-		-	-				26.43			32.81	23.08	23.53	33.94
	Yes	-			-	-		-	-				72.34			67.19	75.6	74.12	63.18
Ibuprofen, %																			
	No	-			-	-		-	-				42.83			52.34	46.68	48.24	53.43
	Yes	-			-	-		-	-				55.59			46.88	51.46	49.8	43.68
Antacid, %																			
	No	-			-	-		-	-				67.66			69.53	52.25	60.78	62.09
	Yes	-			-	-		-	-				30.27			30.47	45.36	35.69	33.94

* Percentages do not sum to 100 because of missing data

 The fully adjusted results of our analyses are shown in Table 3 with nominally statistically significant (p<0.05) results in bold font. From the baseline questionnaire, current heavy work was associated with an increased risk of GCA but there was no trend with less active daily routines, all compared with all-day sitting. Strenuous physical activity in the last 12 months showed tentative evidence of inverse associations with risk for each cancer type, but this was only statistically significant for GNCA. It appeared that a protection threshold was reached by 1–3 times per month (odds ratio (OR)=0.64, 95% confidence interval (CI): 0.42, 0.98) because greater frequency of strenuous physical activity did not further reduce the risk estimate. The results for typical physical activity and sports during ages 15–18 years displayed similar types of associations relative to those of strenuous physical activity in the last 12 months, although the only nominally statistically significant p value was the p for trend for GNCA. Also in tentative support of an inverse association between physical activity and GNCA, were the risk factor questionnaire analyses of recreational moderate-vigorous physical activity at various ages, although only three p values of such were statistically significant. Recent (last ten years) recreational moderate-vigorous physical activity was not associated with any of the cancers assessed and even estimates for GNCA were less indicative of an association. Perhaps surprisingly, increased sedentary behavior―using the proxy question ‘typical number of hours per day watching TV in the last 12 months’―was inversely associated with EA risk. This relationship was not evident for other cancer sites assessed in this analysis. There appeared to be no association between number of hours per day sitting in the last 12 months and future cancer risk. Once associations for each cancer were adjusted for multiple testing [9], none of the results shown in Table 3 were deemed to be statistically significant. Exclusion of BMI, or addition of waist:hip ratio, to the statistical models did not materially alter any of these risk estimates (results not shown). Stratification of analyses by BMI (<25, 25–<30, ≥30) did not present any evidence for effect-modification (results not shown). In the lag analysis, the inverse association between strenuous physical activity and GNCA was attenuated when the first 1/3 and first 2/3s of cancer diagnoses were excluded as events (Tables S1 and S2), although the confidence intervals were wide and encompassed the estimates from the main analysis. Conversely, the inverse association with recreational moderate-vigorous physical activity at ages 35–39 years appeared to strengthen. The only other result to change in the lag analysis was the association between recreational moderate-vigorous activity and EA which became progressively stronger, although HR estimates were ~2.10 for every level of exposure level compared with never in the analysis which excluded the first 2/3s of EA diagnoses as events. Mutually adjusted models had minimal effect on the point estimates ascertained (Table S3).

**Table 3:** Multivariable Cox porportional hazards regression results of the associations between physical activity and sedentary behavior in relation to esophageal and gastric cancers.

**Variable**	**Exposure Level**	**ESCC**			**EA**			**GCA**			**GNCA**	
		**HR (95%CI)**	**P Value**		**HR (95%CI)**	**P Value**		**HR (95%CI)**	**P Value**		**HR (95%CI)**	**P Value**
***Baseline Questionnaire***												
**Current daily routine at work**	All day sitting	*Referent*			*Referent*			*Referent*			*Referent*	
	Mostly sitting	1.08 (0.63, 1.84)	0.78		0.89 (0.65, 1.20)	0.43		1.18 (0.80, 1.75)	0.40		0.90 (0.62, 1.31)	0.58
	Walking, minimal lifting	0.91 (0.53, 1.55)	0.73		0.83 (0.61, 1.12)	0.22		1.27 (0.86, 1.88)	0.23		1.02 (0.71, 1.47)	0.92
	Lift light loads, climb stairs	0.73 (0.40, 1.35)	0.32		0.90 (0.65, 1.26)	0.54		1.02 (0.66, 1.59)	0.92		0.69 (0.45, 1.05)	0.08
	Heavy work	0.73 (0.27, 2.01)	0.55		0.60 (0.34, 1.07)	0.08		**1.77 (1.01, 3.09)**	**0.05**		0.98 (0.54, 1.78)	0.95
***p-trend***			*0.09*			*0.25*			*0.43*			*0.26*
**Strenuous physical activity during last 12 months**	Never	*Referent*			*Referent*			*Referent*			*Referent*	
	Rarely	0.76 (0.42, 1.37)	0.36		0.83 (0.54, 1.26)	0.38		0.66 (0.42, 1.03)	0.07		0.76 (0.51, 1.14)	0.18
	1–3 times/month	0.62 (0.33, 1.16)	0.13		0.77 (0.50, 1.18)	0.23		0.76 (0.49, 1.19)	0.23		**0.64 (0.42, 0.98)**	**0.04**
	1–2 times/week	0.60 (0.33, 1.08)	0.09		0.86 (0.57, 1.28)	0.45		0.72 (0.47, 1.10)	0.13		**0.64 (0.43, 0.94)**	**0.02**
	3–4 times/week	0.73 (0.41, 1.29)	0.28		0.87 (0.59, 1.30)	0.50		0.68 (0.44, 1.04)	0.07		**0.60 (0.41, 0.89)**	**0.01**
	>5 times/week	0.84 (0.47, 1.52)	0.56		0.74 (0.49, 1.12)	0.15		0.71 (0.46, 1.10)	0.12		**0.58 (0.39, 0.88)**	**0.01**
***p-trend***			*1.00*			*0.47*			*0.41*			***0.01***
**Typical physical activity and sports during ages 15–18 years**	Never	*Referent*			*Referent*			*Referent*			*Referent*	
	Rarely	0.49 (0.19, 1.27)	0.14		0.57 (0.28, 1.14)	0.11		0.68 (0.31, 1.49)	0.34		0.82 (0.43, 1.60)	0.57
	1-3 times/month	0.48 (0.18, 1.29)	0.15		0.89 (0.45, 1.76)	0.74		0.81 (0.37, 1.76)	0.59		0.83 (0.42, 1.65)	0.60
	1-2 times/week	0.55 (0.23, 1.33)	0.19		0.60 (0.31, 1.16)	0.13		0.53 (0.25, 1.12)	0.10		0.81 (0.43, 1.53)	0.51
	3-4 times/week	0.49 (0.21, 1.15)	0.10		0.63 (0.33, 1.19)	0.15		0.66 (0.32, 1.36)	0.26		0.74 (0.39, 1.37)	0.33
	>5 times/week	0.53 (0.23, 1.23)	0.14		0.57 (0.30, 1.07)	0.08		0.67 (0.33, 1.37)	0.28		0.62 (0.34, 1.15)	0.13
***p-trend***			*0.75*			*0.07*			*0.74*			***0.01***
***Risk Factor Questionnaire***												
**Typical recreational moderate-vigorous physical activity during ages 15–18 years**	Never	*Referent*			*Referent*			*Referent*			*Referent*	
	<1 hour/week	0.75 (0.30, 1.85)	0.53		0.81 (0.43, 1.53)	0.51		0.63 (0.29, 1.38)	0.25		0.81 (0.46, 1.43)	0.46
	1-3 hours/week	0.96 (0.50, 1.85)	0.91		1.03 (0.65, 1.64)	0.90		0.76 (0.44, 1.33)	0.34		0.80 (0.52, 1.24)	0.32
	4-7 hours/week	0.74 (0.38, 1.44)	0.38		1.22 (0.79, 1.90)	0.37		0.98 (0.58, 1.63)	0.92		0.79 (0.52, 1.21)	0.28
	>7 hours/week	0.77 (0.42, 1.39)	0.38		1.08 (0.71, 1.63)	0.73		1.11 (0.70, 1.77)	0.66		**0.61 (0.41, 0.91)**	**0.01**
***p-trend***			*0.34*			*0.46*			*0.11*			***0.01***
**Typical recreational moderate-vigorous physical activity during ages 19–29 years**	Never	*Referent*			*Referent*			*Referent*			*Referent*	
	<1 hour/week	1.14 (0.45, 2.89)	0.78		0.97 (0.55, 1.71)	0.91		0.83 (0.42, 1.62)	0.59		0.83 (0.48, 1.43)	0.50
	1-3 hours/week	1.47 (0.70, 3.09)	0.31		1.18 (0.75, 1.85)	0.48		1.05 (0.63, 1.77)	0.85		0.76 (0.49, 1.18)	0.23
	4-7 hours/week	1.11 (0.52, 2.34)	0.79		1.48 (0.96, 2.28)	0.07		1.24 (0.76, 2.03)	0.40		0.76 (0.49, 1.16)	0.20
	>7 hours/week	1.17 (0.57, 2.41)	0.67		1.02 (0.66, 1.57)	0.95		1.00 (0.61, 1.64)	0.99		0.73 (0.48, 1.11)	0.14
***p-trend***			*0.96*			*0.83*			*0.71*			*0.17*
**Typical recreational moderate-vigorous physical activity during ages 35–39 years**	Never	*Referent*			*Referent*			*Referent*			*Referent*	
	<1 hour/week	1.42 (0.57, 3.54)	0.45		0.87 (0.54, 1.40)	0.57		0.91 (0.53, 1.56)	0.73		0.60 (0.36, 1.03)	0.07
	1-3 hours/week	1.82 (0.84, 3.94)	0.13		1.01 (0.69, 1.48)	0.97		0.86 (0.55, 1.35)	0.51		0.72 (0.48, 1.07)	0.10
	4-7 hours/week	1.52 (0.70, 3.31)	0.29		1.13 (0.77, 1.64)	0.53		1.00 (0.64, 1.56)	0.99		**0.66 (0.44, 0.99)**	**0.04**
	>7 hours/week	1.59 (0.73, 3.43)	0.24		0.92 (0.62, 1.36)	0.67		0.92 (0.58, 1.44)	0.71		0.75 (0.50, 1.12)	0.16
***p-trend***			*0.46*			*0.94*			*0.98*			*0.33*
**Typical recreational moderate-vigorous physical activity in the last ten-years**	Never	*Referent*			*Referent*			*Referent*			*Referent*	
	<1 hour/week	1.30 (0.68, 2.47)	0.43		0.92 (0.61, 1.39)	0.69		1.31 (0.82, 2.11)	0.26		0.71 (0.43, 1.19)	0.19
	1-3 hours/week	0.85 (0.48, 1.53)	0.59		0.97 (0.69, 1.36)	0.86		1.14 (0.75, 1.72)	0.55		0.90 (0.61, 1.32)	0.59
	4-7 hours/week	0.98 (0.55, 1.73)	0.94		1.05 (0.75, 1.47)	0.77		1.15 (0.76, 1.75)	0.51		0.92 (0.63, 1.36)	0.68
	>7 hours/week	0.88 (0.49, 1.58)	0.66		0.98 (0.69, 1.39)	0.91		1.00 (0.65, 1.56)	0.99		0.83 (0.56, 1.24)	0.36
***p-trend***			*0.50*			*0.84*			*0.81*			*0.65*
**Typical number of hours per day watching TV during last 12 months**	<1 hour/day	*Referent*			*Referent*			*Referent*			*Referent*	
	1-2 hours/day	0.98 (0.43, 2.23)	0.97		0.65 (0.42, 1.01)	0.05		0.93 (0.47, 1.84)	0.83		1.05 (0.56, 1.94)	0.89
	3-4 hours/day	0.79 (0.35, 1.78)	0.57		**0.55 (0.36, 0.84)**	**0.01**		1.32 (0.69, 2.53)	0.41		1.19 (0.65, 2.17)	0.57
	5-6 hours/day	1.21 (0.52, 2.85)	0.66		**0.57 (0.36, 0.92)**	**0.02**		1.23 (0.61, 2.48)	0.57		0.99 (0.51, 1.92)	0.98
	>7 hours/day	0.78 (0.26, 2.32)	0.66		0.55 (0.29, 1.01)	0.05		1.36 (0.60, 3.06)	0.46		0.94 (0.42, 2.11)	0.88
***p-trend***			*0.88*			*0.09*			*0.11*			*0.85*
**Typical number of hours per day sitting during last 12 months**	<3 hours/day	*Referent*			*Referent*			*Referent*			*Referent*	
	3-4 hours/day	0.81 (0.47, 1.39)	0.44		1.06 (0.78, 1.45)	0.70		0.70 (0.49, 1.01)	0.06		1.10 (0.78, 1.56)	0.57
	5-6 hours/day	1.10 (0.66, 1.83)	0.72		1.11 (0.82, 1.51)	0.51		0.79 (0.55, 1.13)	0.20		0.97 (0.68, 1.40)	0.88
	7-8 hours/day	1.03 (0.56, 1.90)	0.92		1.03 (0.71, 1.49)	0.88		0.67 (0.43, 1.05)	0.08		1.05 (0.68, 1.61)	0.83
	>9 hours/day	0.87 (0.40, 1.90)	0.72		0.69 (0.41, 1.15)	0.15		1.00 (0.62, 1.61)	1.00		0.82 (0.46, 1.47)	0.51
***p-trend***			*0.78*			*0.36*			*0.63*			*0.55*

## Discussion

In this analysis of the AARP Diet and Health Study, we find limited evidence for the importance of physical activity and sedentary behavior with respect to risk of esophageal and gastric malignancies. There was evidence for inverse associations between physical activity and GNCA risk, and sedentary behavior and EA. Once adjusted for multiple testing, none of the associations remained statistically significant.

 There have been at least 16 previous studies of physical activity in relation to esophageal cancer [5,10-24], eight of which were cohort studies [5,11,12,14,16,18,19,21] and a further nine of which assessed histology-specific associations [5,10,13-15,17,21,22,24]. Studies which have assessed all esophageal cancer histologies combined have generally been null for associations with physical activity [12,19,20] and sedentary behavior [19], with only the British Regional Heart Study [18] and a study from Montreal, Canada [23] finding evidence for inverse associations between physical activity and upper digestive (oral/esophagus) malignancies and esophageal cancer, respectively. However, two large occupational analyses conducted in occupationally active adults—the U.S. Agricultural Health Study [11] and the Danish Mail Carriers study [16]—also observed reduced risks of esophageal cancer relative to the general population, consistent with the idea of a protective effect conferred by increased physical activity. 

 For esophageal cancer, histology-specific risks may prove to be more informative, given the starkly different risk-factor profiles as well as incidence trends of adenocarcinoma and squamous cell carcinoma. Seven studies have assessed physical activity in relation to EA [5,10,14,15,17,21,22], with two finding evidence for an inverse association [10,17], and a further three with similar inverse estimates close to the threshold of nominal statistical significance (p<0.05) [5,21,22]. One of these studies was a previous analysis of AARP data. The updated and expanded analysis of AARP data we present herein shows that these associations have attenuated with extended follow-up. Moreover, the extended number of physical activity exposures we were able to assess also does not provide any evidence for an association with risk of EA. All previous analyses, including this AARP analysis, adjusted for BMI as this may be considered a potential confounder given its association with physical activity [25] as well as EA [26]. The predominant causal theory for the positive association between BMI with EA is that obesity increases the propensity for acid reflux via increasing intra-gastric pressure [27], distorting the lower esophageal sphincter [28,29], and increasing the likelihood for hiatal hernia [29,30]. An additional, non-mutually exclusive route of association that has been proposed is that visceral adiposity could affect risk of EA via systemic effects such as inflammatory cytokines or sex steroid hormones [29,31,32]. However, our models without adjustment for BMI were not materially different to the results presented herein. 

EPIC is the only previous study to have assessed sedentary behavior in relation to EA risk and in this analysis they found no evidence for association [14]. Given that our results in this AARP analysis are only tentative and not statistically significant after adjustment for multiple testing, it is unlikely that sedentary behavior affects risk of EA. In addition, the results for sedentary behavior are not internally consistent with the results for physical activity, with both TV watching and strenuous physical activity demonstrating inverse relationships with this malignancy. As such, these findings may be due to chance.

There are only two previous studies of physical activity and ESCC [5,13]. The study by Leitzmann et al [5] was an analysis of a previous AARP dataset restricted to the second question of Table 1 from the baseline questionnaire. They presented similar null findings. Etemadi et al [13] presented results from the Golestan Case–Control Study based in Northern Iran, which included 300 ESCC cases and 571 controls. They found inverse associations between physical activity at ages 15 and 30 years and ESCC risk in women, but not in men. Lastly, a recently published study of 703 ESSC cases in India found a positive association with physical activity, the level of which was based on occupation [24]. In summary, the evidence for any association between physical activity and ESCC is weak. There are no previous studies of sedentary behavior and ESCC and our results from AARP are not indicative of an association.

There are 21 publications from 18 previous studies that have assessed physical activity and/or sedentary behavior in relation to gastric cancer/adenocarcinoma [11,12,16,18-21,33-45]. Of the eleven cohort studies to assess physical activity in relation to GC risk [11,12,18-21,39-45], 8 found evidence in favor of an inverse relationship [11,12,18,21,39-41,44], two studies were null [19,20,42], and one study presented tentative evidence for a positive association [43,45]. In addition, two [33,35] of three case-control studies [33-35], and one [36] of four [16,36-38] occupational cohort studies, found evidence in favor of an inverse association, with one occupation cohort finding evidence for a positive relationship, albeit restricted to social class III of five possible groups [38]. The overall impression from these studies of physical activity and GC is one of a potential inverse association, while some of the heterogeneity in results may be explained by both imprecise exposure ascertainment [5] and variable proportions of cardia and noncardia malignancies in the case-groups assessed. The latter is supported by the studies that have conducted site-specific analyses—four [5,14,22,44] of the five studies [5,14,17,22,44] to assess GNCA found evidence of inverse associations between physical activity and cancer risk, while none of the four studies to assess GCA presented evidence of association [5,14,15,17]. These results are in agreement with the estimates we present herein. Only one previous study assessed sedentary work in relation to GC—a large, all-female occupation cohort identified from Finnish census data—and this study found no evidence for association [19], as per our analyses here.

 Strengths of this analysis include the prospective collection of exposure information and cancer outcomes, which reduces the possibility of recall bias affecting the results. Also, the rich exposure history provided by the NIH-AARP study enabled adjustment for many potential confounding factors including BMI, fruit and vegetable consumption, calorie consumption, antacids (as a proxy for gastroesophageal reflux), and socioeconomic status. Lastly, the detailed exposure ascertainment of physical activity and sedentary behavior enabled for a fairly comprehensive analysis, albeit with exposures being self-reported and without validation. Limitations include: not knowing the *Helicobacter pylori* status of cases and controls, although this is unlikely to be a confounding factor given that it has been shown not to be associated with physical activity [46]; the possibility of inaccurate recall given the elapsed time between exposure and time of questionnaire, which in most cases biases results towards the null [47]; and the possibility of misclassification and dilution of any mediated effect, with increasing time between age of exposure ascertainment and cancer diagnosis.

 In summary this analysis of NIH-AARP data presents evidence for inverse associations between physical activity and GNCA risk, a relationship which is supported by previous literature.

## Supporting Information

Table S1 **Multivariable Cox proportional hazards regression results of the associations between physical activity and sedentary behavior in relation to esophageal and gastric cancers.** LAG ANALYSIS—the first 33% of cancer cases were right-censored at date of cancer diagnosis, as opposed be being counted as an event.
(XLS)

Table S2 **Multivariable Cox proportional hazards regression results of the associations between physical activity and sedentary behavior in relation to esophageal and gastric cancers.** LAG ANALYSIS—the first 66% of cancer cases were right-censored at date of cancer diagnosis, as opposed be being counted as an event.
(XLS)

Table S3 **Multivariable Cox proportional hazards regression results of the associations between physical activity and sedentary behavior in relation to esophageal and gastric cancers—mutually adjusted models.**
(XLS)
